# Has the NTD Community Neglected Evidence-Based Policy? *PLOS NTDs* 2013 Expert Commentary of the Viewpoint by Nagpal S, Sinclair D, Garner P

**DOI:** 10.1371/journal.pntd.0002299

**Published:** 2013-07-11

**Authors:** Antonio Montresor, Albis F. Gabrielli, Dirk Engels, Denis Daumerie, Lorenzo Savioli

**Affiliations:** Department of Control of Neglected Tropical Diseases, World Health Organization, Geneva, Switzerland; Lindsley F. Kimball Research Institute, New York Blood Center, United States of America

In July 2013, *PLOS Neglected Tropical Diseases* will publish the viewpoint of members of the Cochrane Infectious Diseases Group [1] on the scientific evidence supporting the public-health benefits of preventive chemotherapy [2] in the case of programmes against neglected tropical diseases (NTDs), specifically those for the control and elimination of lymphatic filariasis, onchocerciasis, schistosomiasis, and soil-transmitted helminthiases. This viewpoint presents the group's interpretation of its findings on health interventions using Cochrane systematic reviews of preventive chemotherapy interventions published in recent years, which do not confirm the benefits of the intervention at the community level [3], [4].

However, these reviews were conducted by analyzing data exclusively from randomized controlled trials (RCTs). WHO considers RCTs study designs that have been developed to compare drug efficacy over short-term periods and that are not appropriate for evaluating interventions such as preventive chemotherapy, which should be applied to a population for at least five consecutive years [5].

The main reason for this is that it is almost impossible to organize proper RCTs for preventive chemotherapy interventions because of ethical reasons (it is not ethically acceptable to exclude infected populations from treatment for five years). In addition, the results of RCTs are analyzed on the assumption that because preventive chemotherapy is applied at population level, its benefits should be observed in the entire population; by doing so, the key point is missed: that this strategy is used not because it will benefit the uninfected, but because it is more cost-effective to treat everyone than to select those who are infected [6].

In our view, the limitations of RCTs (and reviews based exclusively on their analysis to conclude on public-health interventions) are proven by the fact that they consistently confirmed the efficacy of preventive chemotherapy medicines (albendazole, mebendazole, praziquantel, ivermectin, and DEC) against the parasites targeted (because RCTs are designed to evaluate such results) but failed to demonstrate their impact on communities (because RCTs are not designed to measure such impacts).

We consider the best way to evaluate the benefits of preventive chemotherapy interventions to be through monitoring the impact of a disease control or elimination programme for several years. Whenever long-term interventions are evaluated, the impact of preventive chemotherapy on morbidity is evident. For example, Ramzy et al. [7] showed that five annual rounds of mass drug administration eliminated lymphatic filariasis in endemic communities in Egypt; Traoré et al. [8] proved that elimination of onchocerciasis with ivermectin is technically feasible in hyperendemic foci from Mali and Senegal; Sinoun et al. [9] demonstrated the complete elimination of the morbidity caused by schistosomiasis in Cambodia after an eight-year intervention; and Casey et al. [10] showed successful control of soil-transmitted helminthiases and improved haemoglobin levels after 56 months of activities in Viet Nam. Unfortunately, none of these articles merited attention by the authors of this viewpoint, probably because they are not RCTs. We therefore consider the conclusion of the authors of this viewpoint the consequence of a selection bias.

Some studies demonstrated that preventive chemotherapy is beneficial not only for the populations targeted but also for future generations: for example, Ottesen et al. [11] calculated that, between 2000 and 2007, mass drug administration prevented lymphatic filariasis in 6.6 million newborns, thus averting in their lifetimes nearly 1.4 million cases of hydrocele, 800,000 cases of lymphoedema, and 4.4 million cases of subclinical disease. In addition, evidence demonstrates that preventive chemotherapy is beneficial for diseases that are not included in WHO's list of neglected tropical diseases, such as scabies, which benefit from the large-scale application of ivermectin [12], and to mortality in general, which was significantly reduced in Ethiopia by the large-scale distribution of azithromycin [13].

Finally, it seems to us ethically unacceptable that infected human populations are not administered medication that is freely available while livestock and companion animals are regularly dewormed every year.
